# Integrating Computation and Experiment to Investigate Photoelectrodes for Solar Water Splitting at the Microscopic Scale

**DOI:** 10.1021/acs.accounts.1c00418

**Published:** 2021-10-07

**Authors:** Wennie Wang, Andjela Radmilovic, Kyoung-Shin Choi, Giulia Galli

**Affiliations:** † Pritzker School of Molecular Engineering, University of Chicago, Chicago, Illinois 60637, United States; ‡ Department of Chemistry, University of Wisconsin-Madison, Madison, Wisconsin 53706, United States; § Department of Chemistry, University of Chicago, Chicago, Illinois 60615, United States; ∥ Materials Science Division and Center for Molecular Engineering, Argonne National Laboratory, Lemont, Illinois 60439, United States

## Abstract

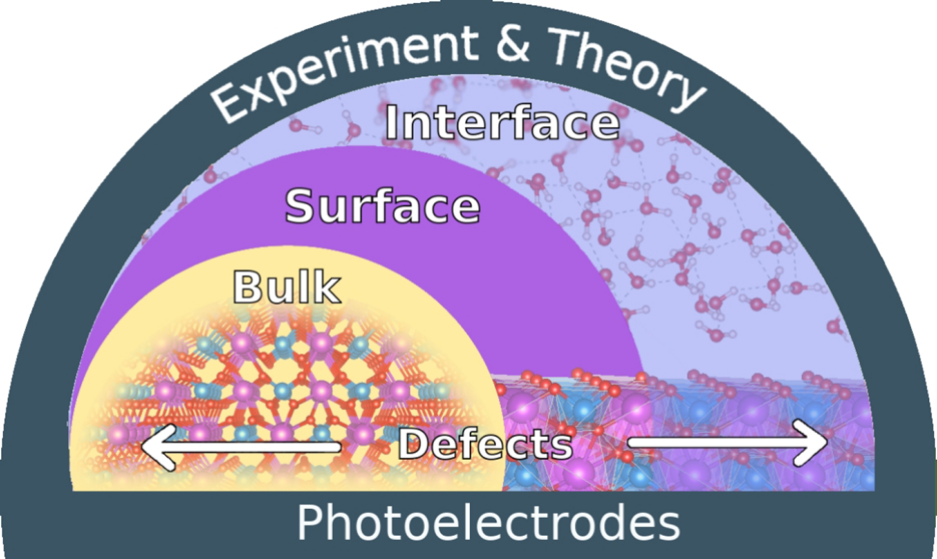

Photoelectrochemical water-splitting is a promising and sustainable way to store the energy of the sun in chemical bonds and use it to produce hydrogen gas, a clean fuel. The key components in photoelectrochemical cells (PECs) are photoelectrodes, including a photocathode that reduces water to hydrogen gas and a photoanode that oxidizes water to oxygen gas. Materials used in photoelectrodes for PECs must effectively absorb sunlight, yield photogenerated carriers, and exhibit electronic properties that enable the efficient shuttling of carriers to the surface to participate in relevant water-splitting reactions. Discovering and understanding the key characteristics of optimal photoelectrode materials is paramount to the realization of PEC technologies.

Oxide-based photoelectrodes can satisfy many of these materials requirements, including stability in aqueous environments, band edges with reasonable alignment with the redox potentials for water splitting, and ease of synthesis. However, oxide photoelectrodes generally suffer from poor charge transport properties and considerable bulk electron–hole separation, and they have relatively large band gaps. Numerous strategies have been proposed to improve these aspects and understand how these improvements are reflected in the photoelectrochemical performance. Unfortunately, the structural and compositional complexity of multinary oxides accompanied by the inherent complexity of photoelectrochemical processes makes it challenging to understand the individual effects of composition, structure, and defects in the bulk and on the surface on a material’s photoelectrochemical properties. The integration of experiment and theory has great potential to increase our atomic-level understanding of structure–composition–property relationships in oxide photoelectrodes.

In this Account, we describe how integrating experiment and theory is beneficial for achieving scientific insights at the microscopic scale. We highlight studies focused on understanding the role of (i) bulk composition via solid-state solutions, intercalation, and comparison with isoelectronic compounds, (ii) dopants for both the anion and cation and their interactions with oxygen vacancies, and (iii) surface/interface structure in the photocurrent generation and photoelectrochemical performance in oxide photoelectrodes. In each instance, we outline strategies and considerations for integrating experiment and theory and describe how this integration led to valuable insights and new directions in uncovering structure–composition–property relationships. Our aim is to demonstrate the unique value of combining experiment and theory in studying photoelectrodes and to encourage the continued effort to bring experiment and theory in closer step with each other.

## Key References

- LindbergA. E.; WangW.; ZhangS.; GalliG.; ChoiK.-S.Can a PbCrO_4_ Photoanode Perform as Well as Isoelectronic BiVO_4_?ACS Appl. Energy Mater.2020, 3, 8658–8666.^1^*By comparing the electronic structure and defect formation in isoelectronic compounds, useful guidelines to develop high-performance oxide-based photoanode are identified.*
- KimT. W.; PingY.; GalliG. A.; ChoiK.-S.Simultaneous Enhancements in Photon Absorption and Charge Transport of Bismuth Vanadate Photoanodes for Solar Water Splitting. Nat. Commun.2015, 6, 87692649898410.1038/ncomms9769PMC4640143.^2^*Nitrogen doping in conjunction with oxygen vacancies is shown to lead to simultaneous enhancement of optical absorption and charge transport in BiVO*_4_.
- GovindarajuG. V.; MorbecJ. M.; GalliG. A.; ChoiK.-S.Experimental and Computational Investigation of Lanthanide Ion Doping on BiVO_4_ Photoanodes for Solar Water Splitting. J. Phys. Chem. C2018, 122, 19416–19424.^3^*The combined effect of cationic doping on the Bi*^*3+*^*site with species of the lanthanide series and oxygen vacancies is found to be critical to understanding the changes in charge transport in BiVO*_4_*photoanodes.*
- LeeD.; WangW.; ZhouC.; TongX.; LiuM.; GalliG.; ChoiK.-S.The Impact of Surface Composition on the Interfacial Energetics and Photoelectrochemical Properties of BiVO_4_. Nat. Energy2021, 6, 287–294.^4^*As demonstrated with BiVO*_4_*photoanodes, the surface composition plays a critical role in the interfacial energetics and photoelectrochemical performance of the photoelectrode.*

## Introduction

Photoelectrochemical cells (PECs) offer a sustainable and clean approach to converting solar energy into valuable chemical fuels through the use of semiconductor electrodes that absorb sunlight (photoelectrodes). A water-splitting PEC, which produces H_2_ as a clean fuel, is currently the most extensively investigated photoelectrochemical device.^5−9^ An ideal photoelectrode material used in PECs must meet several criteria. It should have a band gap in the visible region where the solar radiation is highest, and it should have a sufficiently large absorption coefficient.^10−12^ The electronic properties of the material must be such that photogenerated electron–hole pairs can be effectively separated and participate in the desired surface chemical reactions^13^ and also have appropriate carrier conductivities.

The search for optimal photoelectrodes is an active field of research and remains a complex task since the intrinsic properties of materials are largely insufficient to determine their promise as photoelectrodes; in fact, defects and impurities are always present at operating conditions and can considerably influence the photon absorption,^12,14^ electron–hole separation, and charge transport properties.^15−17^ Furthermore, the surface composition and morphology of the photoelectrode in aqueous environments and its interfacial properties with other components such as a catalyst layer are usually critical to determining the device performance.^4^

Understanding and controlling the effects of bulk and surface composition, structure, and defects on a material’s photoelectrochemical properties are challenging tasks as the effects are often entangled in multiple intricate processes. Tightly integrated experimental and computational investigations are thus required to deconvolute the effects of many factors and comprehensively understand multiple effects caused by a single factor; such an integration is necessary to gain an atomic-level understanding of structure-composition relationships on the photoelectrochemical properties of materials. Experimentally, it is critical to prepare high-quality samples whose purity, stoichiometry, and structure match the models used in computational studies as closely as possible. When investigating the effect of a specific aspect of the photoelectrode material, only that aspect should be systematically changed to ensure the accurate interpretation of the observed phenomena. For example, to analyze the effect of a dopant, the pristine and doped samples must be prepared under well-controlled synthesis conditions so that the introduction of the dopant changes only the bulk properties and not other factors such as surface area or morphology. Computationally, it is important, and in many cases still challenging, to develop a representative structural model that captures the key features of the system and properties of interest, takes experimental results into account, and uses an appropriate level of theory.^18^ Identifying quantities that can be both measured and simulated under the same or at least similar conditions is critical to enabling a meaningful comparison between experiment and theory.

In this Account, we discuss how combined experimental and theoretical studies can be used to comprehensively understand the structure- and composition-dependent photoelectrochemical properties of photoelectrode materials. We use oxide-based photoelectrodes as examples. These systems have garnered significant interest owing to their relative stability in aqueous environments and ease of synthesis and processing.^8,19−21^ Major bottlenecks of many oxide systems, however, include their relatively wide band gaps and considerable bulk carrier recombination. Thus, it is imperative to combine experimental and computational studies that can provide guidelines to improve the photon absorption and photogenerated charge utilization of oxide photoelectrodes. Ideally, experiment and theory should be integrated through multiple feedback loops to validate and interpret experimental and computational results. Here, we discuss several representative studies of ours that use combined experimental and theoretical approaches to demonstrate how they have elucidated the effects of structure and composition on microscopic processes and macroscopic observables. The new insights gained from these studies greatly enhance our understanding of the photoelectrochemical properties of oxide photoelectrodes, allowing for significant improvement in the future development of PECs.

## The Impact of Bulk Composition

We first discuss three representative cases in which the integration of experiment and theory led us to understand the effect of bulk composition on photoelectrochemical properties. We altered the bulk composition of pristine oxides by forming solid solutions and by intercalation, and we examined the similarities of two isoelectronic oxides.

In the first case, the bulk composition was varied to form solid solutions.^11^ It was experimentally observed that the band gap of a CuWO_4_ photoanode could be effectively decreased by forming solid solutions with CuMoO_4_ (i.e., CuW_1–*x*_Mo_*x*_O_4_). Specifically, CuW_0.35_Mo_0.65_O_4_ (E_g_ = 2.0) showed higher incident photon-to-current conversion efficiencies (IPCEs) than CuWO_4_ (*E*_g_ = 2.3) in the entire visible range for photoelectrochemical water oxidation, indicating that the decrease in the band gap resulted in increased photocurrent generation.

In order to interpret experiments, density functional theory (DFT)^22,23^ calculations were performed to elucidate the effect of solid solution formation on the electronic band structure of CuWO_4_ and thus uncover the microscopic factors responsible for the improved properties of CuW_1–*x*_Mo_*x*_O_4_ compared to CuWO_4_. Different levels of theory^24−26^ (the local density approximation (LDA), the LDA+U approximation, and the generalized gradient approximation (GGA)) were employed to model CuWO_4_, CuMoO_4_, and CuW_1–*x*_Mo_*x*_O_4_. We note that modeling transition metal oxides with DFT generally involves testing and validating a plethora of system-dependent choices including the functional and pseudopotential. Unfortunately, a general DFT-based first-principles method that is appropriate for the description of broad classes of transition metal oxides is not yet available. An open question still is the treatment of strongly correlated electronic states in oxides. A validation procedure that considers the relevant aspects of the atomic structure (e.g., lattice parameters, bond lengths) and electronic structure (e.g., band alignment, charge localization properties, magnetic ground state, band gap) must be performed for each system.^18,27^

In the case of CuWO_4_ and CuMoO_4_, LDA structural parameters were found to be in better agreement (2% error) with experiment compared to GGA (2–3% error). Furthermore, the magnetic ordering, which can affect the lattice parameters and electronic structure, was investigated in detail. The relaxed structure of CuWO_4_ and CuMoO_4_ with antiferromagnetic ordering was better matched (1–2% error) to that of the measured low-temperature structure^28,29^ compared with nonmagnetic structures (3% error). Interestingly, the conclusions of calculations with and without a Hubbard U parameter (LDA+U with U up to 7 eV) were the same. Since the primary goal of the study was not to reach quantitative accuracy but to achieve insights on the effects of chemical doping on the electronic structure, LDA was deemed to be sufficiently accurate. Calculated electronic band structures showed that the conduction band minimum (CBM) of CuWO_4_ is composed of hybridized W 5d and O 2p states, while the valence band maximum (VBM) is mainly composed of Cu 3d and O 2p states. Although there exists a d–d optical transition that is lower in energy, calculations showed the relevant optical transition measured in the UV–vis region is a p–d transition, which may be meaningfully tuned with doping. In general, determining the band gap in oxides is not trivial.^30^ The decreased band gap of CuW_1–*x*_Mo_*x*_O_4_ is mainly due to the lowering of the CBM as W 5d states are replaced by Mo 4d states. As the CBM of CuWO_4_ is already more positive than that of the water reduction potential by a few hundred millivolts, lowering the CBM is not favorable for overall water splitting. Instead, as its VBM is far more positive than the water oxidation potential, the band gap of CuWO_4_ should be lowered by raising the VBM. Calculations suggested that composition tuning of the Cu and O sites would raise the VBM and provide a promising route to improved water-splitting performance of CuWO_4_.

**Figure 1:**
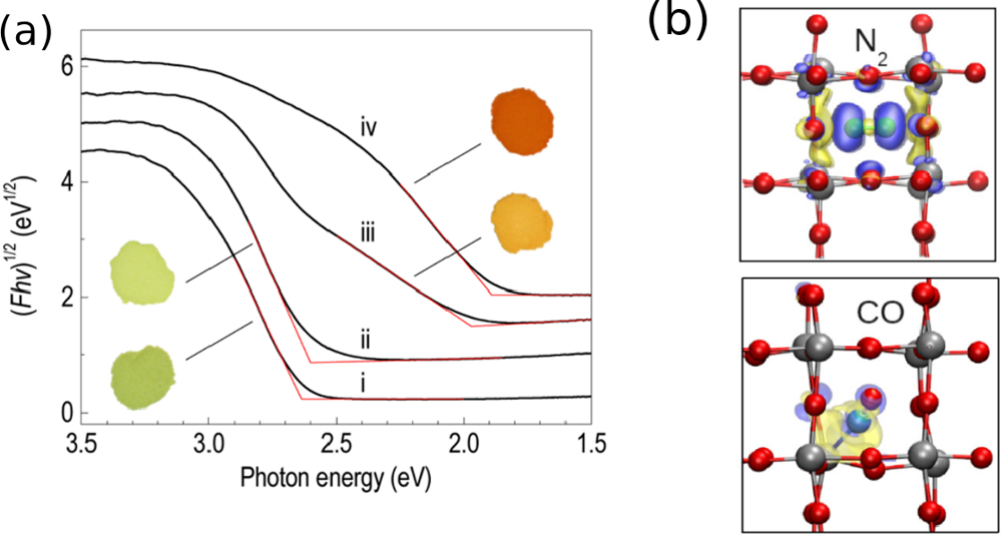
(a) Diffuse reflectance spectra (vertically offset for clarity) and corresponding color of (i) pure monoclinic WO_3_ and ii-iv) WO_3_ containing different amounts of N_2_ prepared at different temperatures. Reproduced from ref (31). Copyright 2012 American Chemical Society. (b) Calculated isosurfaces of electron density differences of monoclinic WO_3_ with N_2_ and CO; yellow corresponds to electron loss, blue to electron gain. Gray atoms are W, red atoms are O. Reproduced from ref (32). Copyright 2012 American Chemical Society.

In the second case, the bulk composition was altered by intercalation.^31^ Tungsten oxide (WO_3_) has a perovskite-type structure (ABO_3_) with vacant A sites. WO_3_ samples with N_2_ intercalated at the A site were experimentally prepared and shown to exhibit smaller band gaps than pristine WO_3_ (Figure 1a). Because one of the fundamental limitations of WO_3_ is its relatively large band gap (∼2.6 eV), this experimental observation warranted detailed investigations of the system. In order to build a meaningful and representative structural model, the amount of N_2_ and the structure of N_2_-intercalated WO_3_ were carefully characterized using various experimental techniques. Specifically, nitrogen was confirmed to be present as N_2_ and not as any other N-species (such as NO or NO_2_), and it was shown to be intercalated in the A site without substitutionally replacing other components.

Using information about experimentally determined structural properties as a starting point, calculations based on DFT (Figure 1b) revealed that the decrease in the band gap of N_2_-intercalated WO_3_ is not caused by the direct interaction of N_2_ states with states at the CBM or VBM of the host material. Instead, it is due to steric changes in the WO_3_ lattice resulting from the intercalated N_2_, leading to an increase in the symmetry of the lattice. The structure of WO_3_ is composed of corner-shared WO_3_ octahedra. Since the CBM of WO_3_ is composed of W 5d–O 2p π* orbitals, the W–O–W bond angle directly affects the CBM position and a higher symmetry lowers the CBM and decreases the band gap.

Calculations based on DFT^12,32^ provide important qualitative insights and key information about trends of material properties as a function of identified descriptors. However, the study of many-body effects on electronic excitations requires the use of higher levels of theory. Therefore, we also performed calculations based on many-body perturbation theory in the Green’s function formalism to solve approximate forms of the Bethe–Salpeter equations (BSE)^33−35^ in order to obtain an accurate, quantitative understanding of optical absorption in WO_3_.^12^ We found that intercalating N_2_ decreases the exciton binding energy between electron–hole pairs in WO_3_, which in turn leads to improved charge separation but weakened absorption near the band edge.^12^ These effects would not have been observable using standard DFT, thus illustrating the value afforded by higher levels of theory for specific materials properties.

In addition to N_2_-intercalation in WO_3_, we investigated other species such as CO and noble gas atoms (Ne, Ar, Xe) that could intercalate into the A site of the host and lead to a decrease in the band gap by raising the VBM.^32^ Notably, DFT calculations showed that CO binds to W in the WO_3_ lattice, and the Coulombic repulsion between the carbon lone-pair electrons and nearby O nonbonding p orbitals leads to an upward shift of the VBM by 0.15 eV compared to that of pristine monoclinic WO_3_. These examples highlight possible strategies for improving photoelectrode performance by modifying the host lattice.

**Figure 2:**
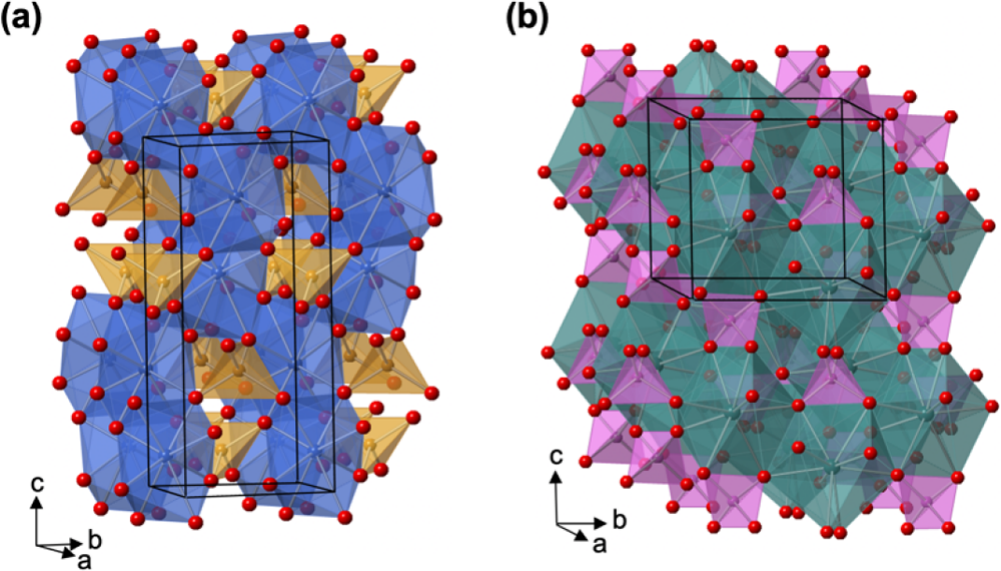
Crystal structures of (a) BiVO_4_ and (b) PbCrO_4_. Bi is blue, V is gold, Pb is green, Cr is pink, and O is red. Reproduced from ref (1). Copyright 2020 American Chemical Society.

The third case involves a comparative study of two related compounds to identify common features responsible for their photoelectrochemical performance. Upon reviewing numerous ternary oxide photoelectrodes,^19^ it became evident that BiVO_4_ has an exceptional electron–hole separation yield that is considerably higher (almost 100% at 1.23 V vs RHE and exceeds 70% at 0.6 V vs RHE) than that of other oxide photoelectrodes (<10% at 1.23 V). This raised the interesting question of whether other compounds that possess similar electronic features to BiVO_4_ can have comparably high electron–hole separation yields. Hence, we studied lead chromate (PbCrO_4_), whose composition and structure closely resemble those of BiVO_4_, and we compared its properties with those of BiVO_4_ (Figure 2).^1^ We note that previous studies of PbCrO_4_ had reported poor electron–hole separation, but PbCrO_4_ electrodes used in those studies were low-quality in terms of purity and uniformity, which might have affected their performance. Therefore, the preparation of high-quality samples was critical to fairly assessing the photoelectrochemical properties.

We prepared a high-purity, uniform PbCrO_4_ photoanode that demonstrated an exceptionally high electron–hole separation yield comparable to that of BiVO_4_; we then performed a computational study to compare the electronic structures of BiVO_4_ and PbCrO_4_. The computational groundwork involved identifying the low-energy surfaces and determining a level of theory appropriate for both PbCrO_4_ and BiVO_4_. DFT+U calculations revealed that these two compounds have similar electronic structures (e.g., VBM and CBM composition) and the high electron–hole separation efficiencies of both compounds indeed appeared to be related to their similarities in electronic and atomic structure (e.g., coordination environments of metal ions, connectivity of polyhedra, and low probability for metal ions swapping sites to form defects). The common features identified between PbCrO_4_ and BiVO_4_ provide useful guidelines for identifying other oxide photoanodes that can achieve high electron–hole separation efficiencies.^1^

## The Role of Dopants and Defects in the Bulk

Considering that poor charge transport and high electron–hole recombination in the bulk are common drawbacks of most oxide photoelectrodes, one of the major efforts in improving their performance has focused on enhancing carrier transport via oxygen vacancies or substitutional dopants. Despite numerous studies, several controversial statements regarding the nature of oxygen vacancies as donors can be found in the literature. In many transition metal oxides, oxygen vacancies appear to be deep donors if their ionization energy is calculated relative to the CBM, a popular convention used to estimate the ionization energy of defects. However, in many oxides, charge transport occurs through polaron hopping, not band transport, and the relevant ionization energy of oxygen vacancies in polaronic oxides should be calculated with respect to a free polaron state, which is the state of the polaron when it is not bound to a defect. This concept, which is critical to understanding the role of oxygen vacancies and dopants in polaronic oxides, was first used to understand the role of oxygen vacancies in determining the bulk conductivity of BiVO_4_.^36^ The following combined experimental and computational studies further justify the use of a free polaron state when calculating the defect ionization energy in polaronic oxides.^37,38^

**Figure 3:**
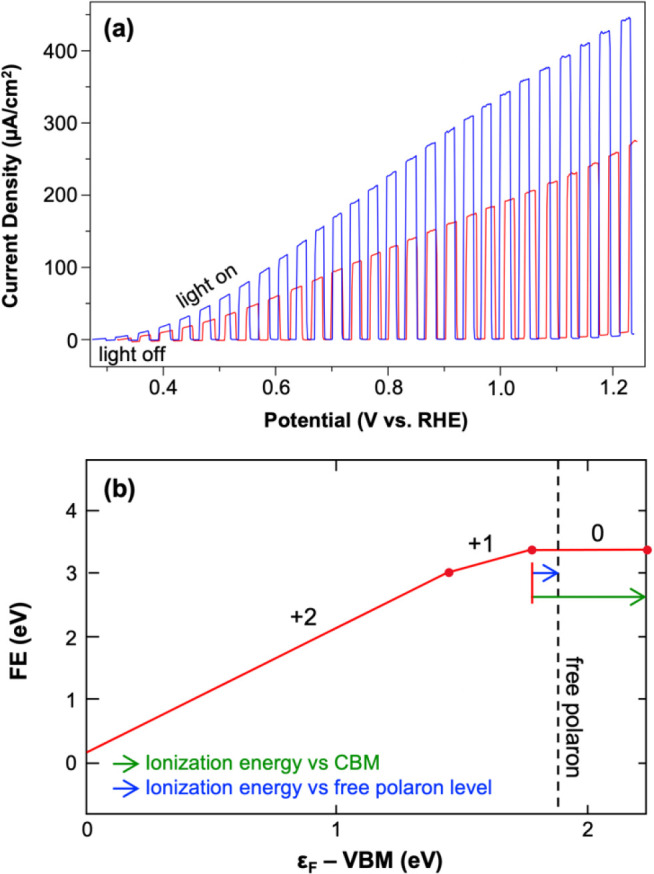
(a) *J*–*V* plots for pristine (red) and N_2_-treated (blue) BiFeO_3_ for sulfite oxidation (pH 9.2 borate buffer) under AM 1.5G illumination; (b) defect formation energy diagram of an oxygen vacancy in BiFeO_3_. Reproduced from ref (37). Copyright 2020 American Chemical Society.

In one study,^37^ high-quality n-type BiFeO_3_ electrodes were prepared and the photoelectrochemical properties of pristine BiFeO_3_ photoanodes were compared with those of N_2_-treated BiFeO_3_, which was expected to contain an increased number of oxygen vacancies. The N_2_-treated BiFeO_3_ showed approximately a 2-fold increase in both photocurrent (Figure 3a) and majority carrier density. This result could not be explained by theoretical studies that considered oxygen vacancies as deep donors with ionization energies greater than 1 eV;^39,40^ the presence of such levels would mean that oxygen vacancies cannot increase the majority carrier density at room temperature. We conducted calculations to confirm that a small electron-polaron forms at an Fe site when an extra electron is added into the pristine BiFeO_3_ system. The ionization energy of an oxygen vacancy, which introduces two electron-polarons, was then calculated relative to the free polaron level (Figure 3b), and the energy difference of the first charge transition level (0/+1) of the oxygen vacancy to the free polaron level was found to be comparable to *kT* at room temperature (26 meV). Approximately 2% of the oxygen vacancies in BiFeO_3_ were estimated to be ionized at room temperature, indicating that they can contribute to the majority carrier concentration. This computational result was consistent with experimental observations, confirming that the ionization energy of an oxygen vacancy in a polaronic oxide should be measured with respect to the free polaron level.

A similar result was obtained when examining substitutional Sn doping in n-type Fe_2_TiO_5_.^38^ First-principles calculations showed that the extra electron gained from substitutional doping of Sn^4+^ at the Fe^3+^ site spontaneously forms a small electron-polaron on the nearest Fe^3+^ site, converting Fe^3+^ to Fe^2+^. Using the conventional definition of ionization energy (i.e., the energy difference between the charge transition level and the CBM), the ionization energy of the Sn dopant would be ∼0.58 eV, indicating that the Sn dopant is a deep donor that cannot increase the carrier density of Fe_2_TiO_5_ at room temperature. However, when the ionization energy is referred to the free polaron level as in the case of BiFeO_3_, it is only ∼0.17 eV, which is several multiples of *kT* at room temperature (26 meV). This result indicates that it is possible for a small fraction of Sn dopants to be ionized at room temperature and enhance the charge transport properties of Fe_2_TiO_5_, a conclusion that agrees well with the experimentally observed photocurrent enhancement caused by Sn doping.

**Figure 4:**
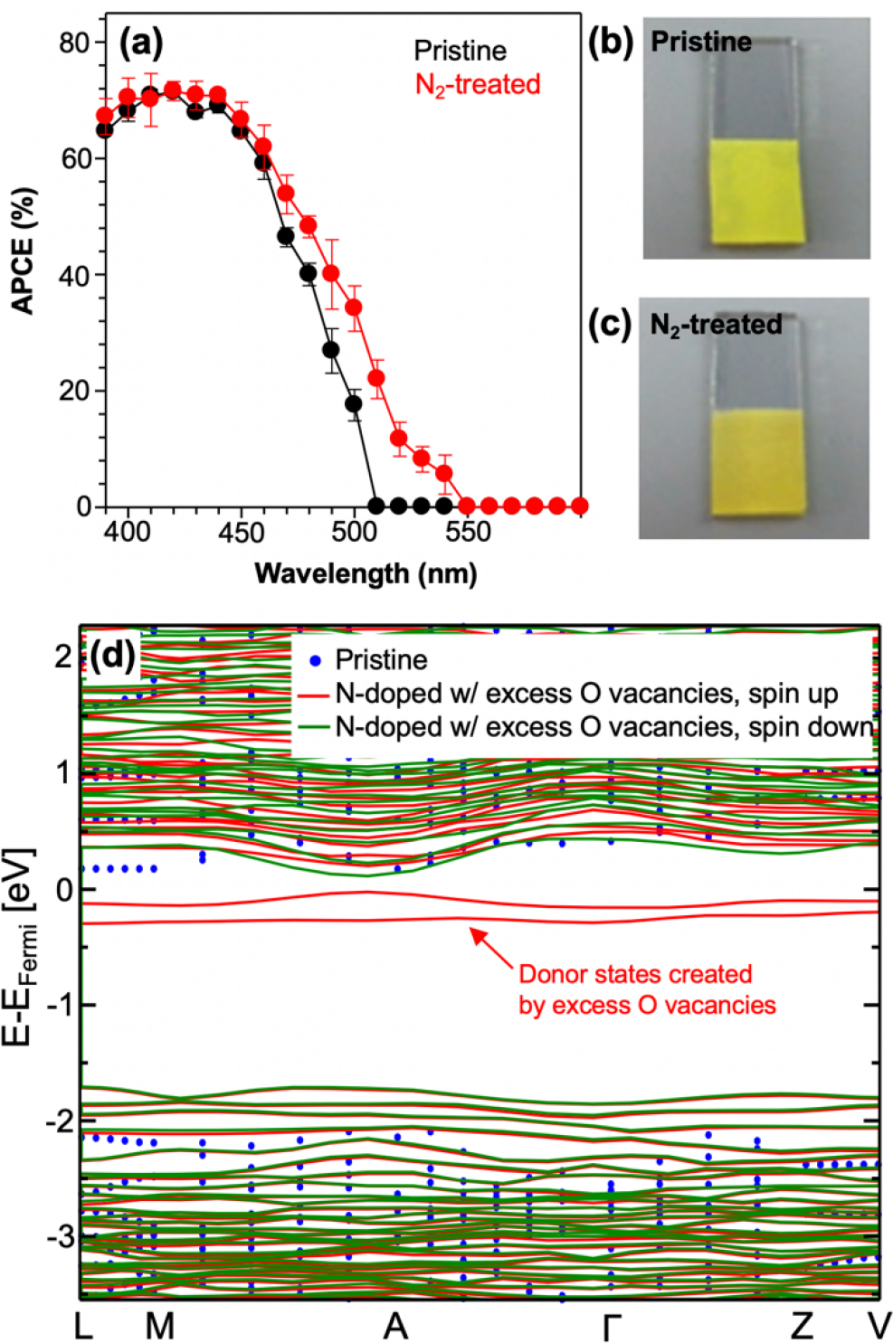
(a) APCE and (b, c) photos of the pristine and N_2_-treated BiVO_4_. APCE was obtained for sulfite oxidation at 0.6 V vs RHE in 0.5 M phosphate buffer (pH 7.2) containing 1 M Na_2_SO_3_ under AM 1.5G illumination. (d) Comparison of band structures of pristine BiVO_4_ (blue dots) and N-doped BiVO_4_ with excess O vacancies with spin up (red) and spin down (green) configurations. Reproduced from ref (2). CC BY 4.0.

We also explored the combined influence of substitutional dopants (for both the anion and cation) and oxygen vacancies on charge transport properties in the case of BiVO_4_. Notably, n-type BiVO_4_ was annealed in a N_2_ environment with the goal of increasing the majority carrier density by increasing the number of oxygen vacancies.^2^ The experimental results revealed that the N_2_ treatment not only created more O vacancies but also resulted in the substitutional doping of N at the O site. These changes enhanced both the photon absorption and electron–hole separation of BiVO_4_ (Figure 4a–c), as evident in the comparison of the absorbed photon-to-current efficiency (APCE) of the pristine and N_2_-treated samples.

DFT calculations helped deconvolute the effects of O vacancy and N incorporation on the photoelectrochemical properties of N_2_-treated BiVO_4_. They revealed that the charge-balanced replacement of O with N (i.e., replacement of three O^2–^ ions with two N^3–^ ions, creating one O vacancy) leads to enhanced photon absorption without forming donor levels. The computational results also showed that if N_2_ treatment generates an increasing number of oxygen vacancies, the latter can form donor levels (Figure 4d) and increase the carrier density. Furthermore, the calculations predicted that the lower static dielectric constant in N-doped BiVO_4_ relative to pristine BiVO_4_ can lead to an enhanced polaron mobility, suggesting an additional mechanism by which charge transport may be improved. Overall, first-principles calculations provided a microscopic explanation of why a simple N_2_ treatment can enhance photon absorption, carrier concentration, and carrier mobility of BiVO_4_.

In addition to N_2_ treatment and its interplay with oxygen vacancies, we investigated the combined influence of oxygen vacancies with substitutional cationic doping at the Bi^3+^ site of BiVO_4_ with various rare earth ions (Ln^3+^: La^3+^, Ce^3+^, Yb^3+^, and Sm^3+^).^3^ Doped samples with the same morphology and surface area as pristine samples were prepared, and the substitutional incorporation of dopants into the BiVO_4_ lattice was confirmed by XRD measurements. While all samples involved the same isovalent replacement of Bi^3+^ with Ln^3+^, the doped samples showed large variation in photocurrent generation. A decrease in photocurrent was observed after La and Ce doping, while an enhancement was observed after Sm and Yb doping. DFT calculations revealed that the decreased photocurrents of La- or Ce-doped samples were due to an increase in the effective masses of electrons and holes upon doping. (For Ce doping, a deep interband state also forms, which may serve as a recombination center.) Furthermore, while Sm or Yb doping alone did not lead to donor states, when combined with oxygen vacancies, donor states arose in the Sm- and Yb-doped samples. These donor states can result in enhanced photocurrent generation. Overall, we found that, in order to interpret the experimental results, it was critical to consider the interaction of Sm or Yb doping with oxygen vacancies and, again, the understanding gained from this study would have not been possible without a combined experimental and computational investigation.

**Figure 5:**
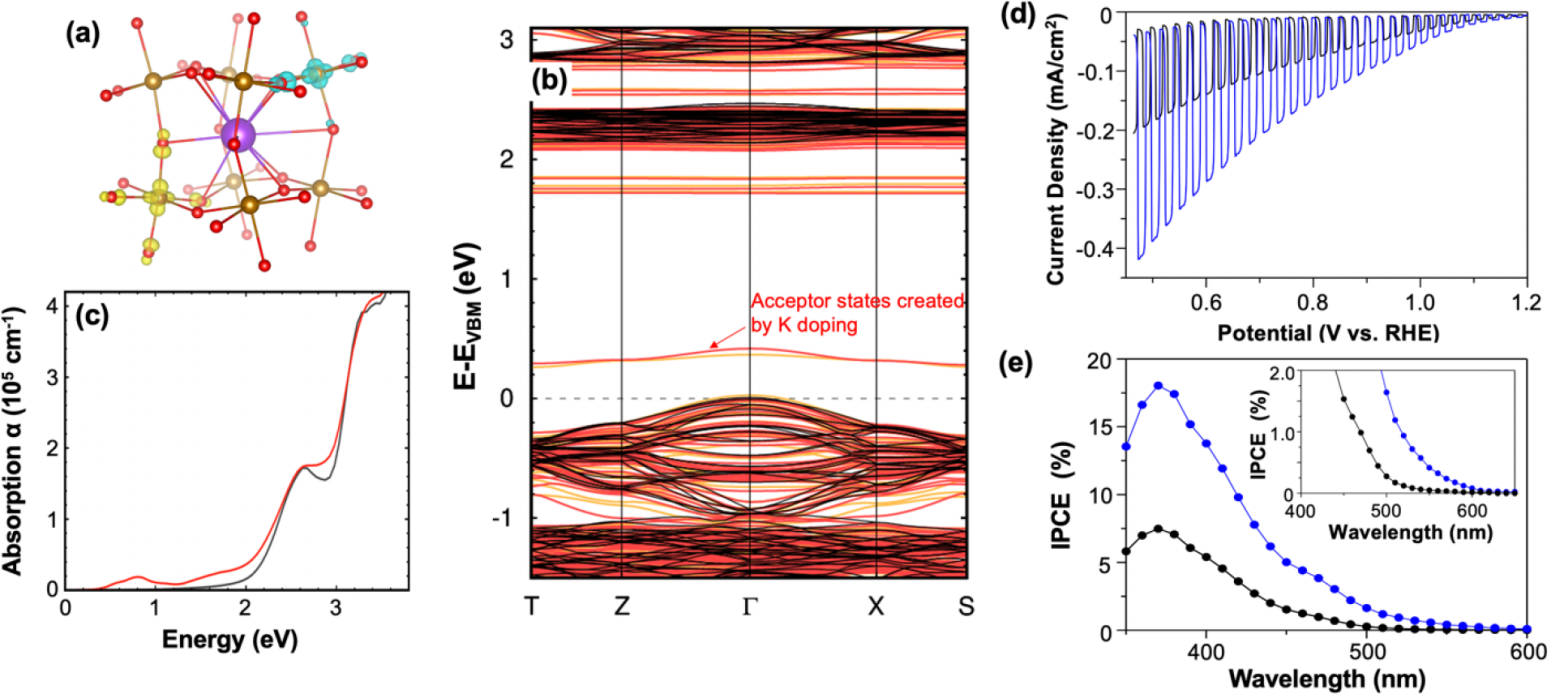
(a) Hole-polaron wave function modulus showing the two holes gained from K doping (purple) localized on nearby Fe sites (brown) and their neighboring O atoms (red) (yellow = spin up; turquoise = spin down); (b) band structure of pristine (black) and K-doped LaFeO_3_ (red = spin up, orange = spin down); (c) calculated absorption spectra for pristine (black) and K-doped (red) LaFeO_3_; (d) *J*–*V* plots (scan rate: 10 mV/s) and (e) IPCE at 0.65 V vs RHE for pristine (black) and 3% K-doped (blue) LaFeO_3_ for oxygen reduction in 0.1 M KOH (pH 13) saturated with O_2_ under AM 1.5G illumination. Reproduced from ref (41). Copyright 2019 American Chemical Society.

We now turn to consider p-type photoelectrodes (photocathodes), for which substitutional doping at the metal site with dopants of lower valency can increase the majority carrier density (holes). A recent combined study investigated substitutional doping of K^+^ at the La^3+^ site in p-type LaFeO_3_.^41^ The computational results showed that, upon K doping, two holes localize on nearby Fe sites and their neighboring O atoms, forming hole polarons and converting Fe^3+^ to Fe^4+^(Figure 5a). Furthermore, the CBM decreased by 0.07 eV and K doping was shown to generate shallow acceptor levels above the VBM (Figure 5b). Calculated absorption spectra (Figure 5c) showed that the changes in electronic structure due to K doping can indeed enhance absorption in the low-energy region (<2.4 eV). Because the solar spectrum contains a considerable number of photons near 2 eV, even a small increase in absorbance in this region can significantly increase the number of photons used by LaFeO_3_ for photocurrent generation. Finally, the calculations showed that K doping generates shallow acceptor levels above the VBM, which can increase the hole concentration of LaFeO_3_.

In order to verify the first-principles predictions, pristine and 3% K-doped LaFeO_3_ of the same morphology were prepared to unequivocally evaluate the effect of K doping.^41^ The experimental results showed increased photon absorption below the band gap of the pristine sample and increased majority carrier density upon K doping, as confirmed by absorption spectra and Mott–Schottky plots, respectively. Furthermore, the Fe^4+^ concentration from the Fe XPS increased, verifying that the additional holes gained from K doping are localized on Fe^3+^ as hole polarons. In terms of photoelectrochemical performance, K-doped LaFeO_3_ showed twice as much photocurrent as pristine LaFeO_3_ (Figure 5d) and IPCE measurements confirmed that both the increased photon absorption and majority carrier density contribute to the photocurrent enhancement (Figure 5e). This combined study provided an atomic-level understanding of how a single dopant can affect both the photon absorption and charge transport properties of a material.

**Figure 6:**
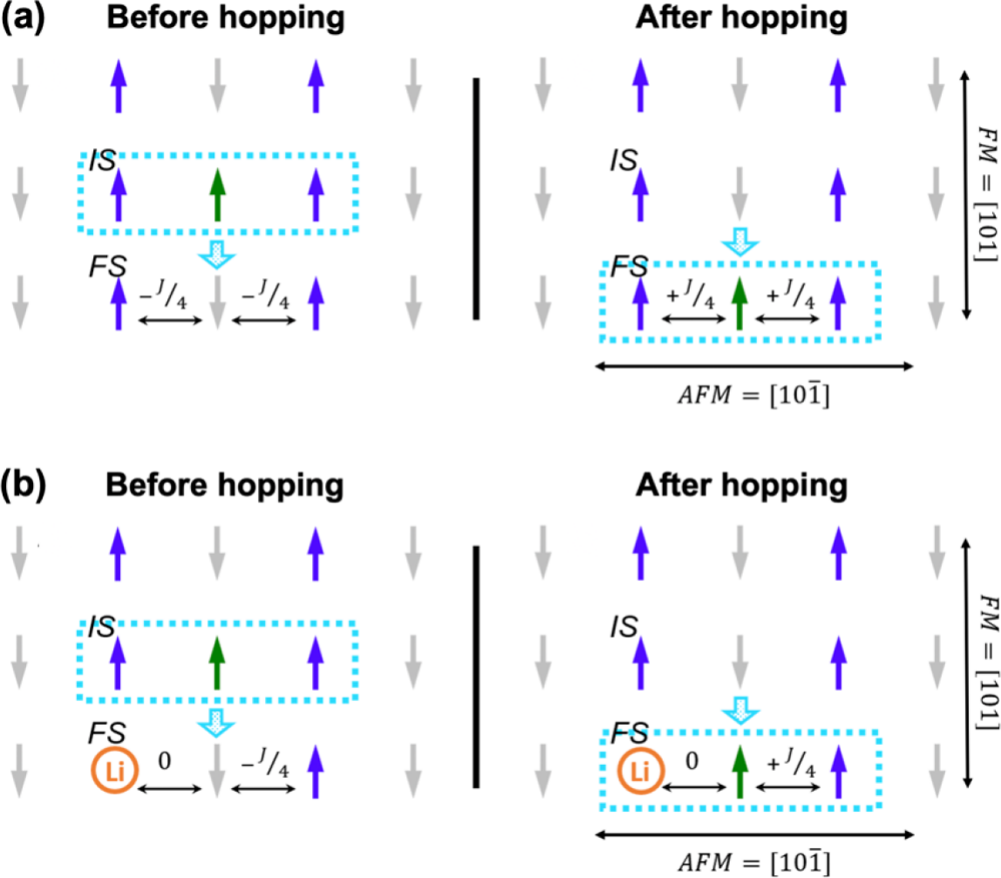
(a) Diagram illustrating how spin-polaron hopping affects the magnetic ordering in pristine CuO. Only Cu spins are shown for simplicity. The spin-polaron forms at the initial site (IS). Before spin-polaron hopping, the spin at the final site is aligned antiferromagnetically (AFM) along [101̅]. Upon hopping, the Cu spin flips and aligns ferromagnetically, which requires energy according to the magnetic coupling constant, *J*. (Blue = Cu with spin up, gray = Cu with spin down, green = Cu with flipped spin, dashed light blue box = polaron state.) (b) The same diagram for the case of CuO after Li doping (orange = Li). Because nonmagnetic Li breaks magnetic couplings between Cu neighbors, the spin-polaron hopping to the Cu site adjacent to Li has a lower magnetic barrier (*E*_a_^spin^). Reproduced from ref (42). CC BY 4.0.

As a final example in this section, we briefly describe the effect of doping on the hole-polaron transport in a magnetic polaronic oxide such as p-type CuO.^42^ A hole in p-type CuO forms a localized polaron state that predominantly contains Cu 3d and O 2p states. Hole localization on a Cu 3d^9^ ion induces a flip in the Cu magnetic moment, forming a spin-polaron. (Flipping a spin can significantly lower the kinetic energy of the state by increasing the delocalization of the hole-polaron.) As a result, the conduction of holes in CuO occurs through spin-polaron hopping that involves a spin-flip process (green arrow in Figure 6a). The activation energy, *E*_a_, for spin-polaron hopping comprises contributions from both the electron–phonon process, *E*_a_^e–ph^, and the spin-flip process, *E*_a_^spin^ (eq 1), resulting in particularly low carrier mobilities in polaronic magnetic oxides.
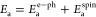
1

Our recent study showed that substitutional doping of Li^+^ at the Cu^2+^ site can improve hopping conduction.^42^ Calculations showed that Li doping creates shallow acceptor states that can increase the hole concentration and simultaneously lower the hopping barrier by reducing both the magnetic (Figure 6b) and electron–phonon couplings, thereby improving the carrier mobility. Indeed, experimentally prepared Li-doped CuO photocathodes showed a significantly enhanced photocurrent compared to CuO photocathodes. This combined study shows that for polaronic magnetic oxides, selecting a nonmagnetic dopant, which can improve both the carrier density and the mobility of spin-polarons, is necessary to effectively improve the photocurrent generation.

## The Impact of Defects and Composition at the Surface/Interface

We now turn to discussing the properties of surfaces and interfaces of oxide photoelectrodes. A typical photoelectrode comprises several components including the photon-absorbing material and a catalyst. The overall properties of the photoelectrode are affected not only by the individual components but also by their interfacial structures, which are challenging to determine and characterize. Experiment can inform theory on the macroscopic impact of surface composition on photoelectrode performance, for which the time and length scales are challenging to simulate computationally. Theory can inform experiment on the relevant surface/interfacial structural motifs at the microscopic scale, which can be difficult to identify unambiguously in experiments; theory can also predict the electronic structure corresponding to given geometric arrangements at the surface. We illustrate this synergy with a recent study on the surface of BiVO_4_, which integrated experiment and theory from start to finish and unambiguously identified the surface composition as a critical factor in photoelectrochemical performance.^4,15^ First, the structure and level of theory for the computed BiVO_4_ surface slabs^15^ were validated with measurements available in the literature on single-crystalline samples that were substrate-free and measured in ultrahigh vacuum.^43^ This made it possible to validate the surface reconstructions, absolute band alignments, and work function of the pristine surface obtained from DFT calculations. We further compared and contrasted the electronic behavior and polaron formation of surface oxygen vacancies with those in the bulk, finding that the oxygen vacancies at the surface are less mobile than those in the bulk,^36^ which could contribute to unwanted carrier recombination.

**Figure 7:**
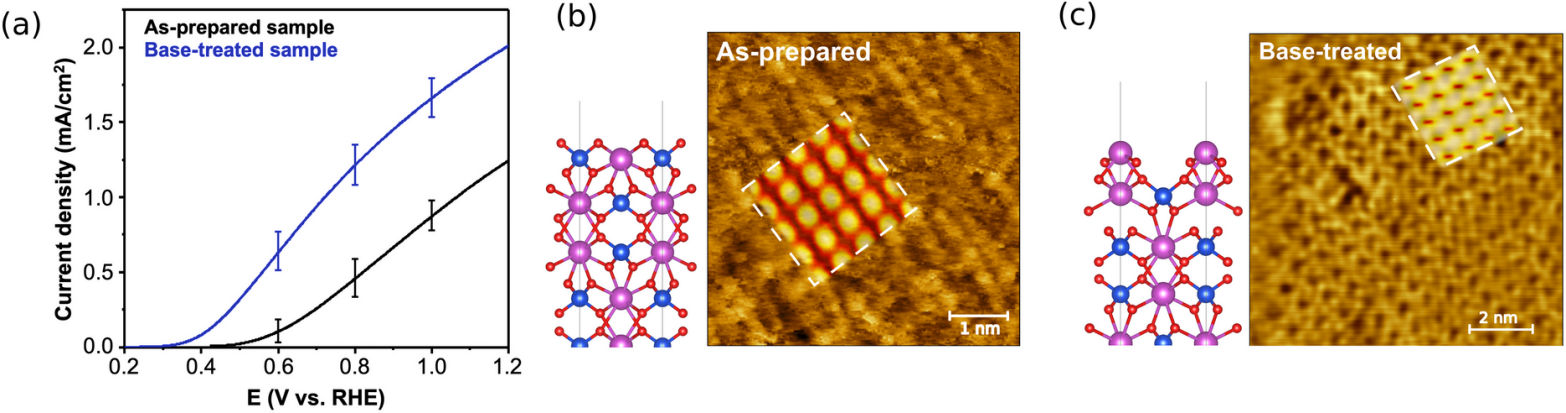
(a) Measured *J*–*V* plots of the as-prepared (black) and base-treated (blue) samples under sulfite oxidation (pH 9.3 borate buffer); error bars represent standard deviations of the current density at selected potentials averaged over six samples. Measured and simulated (dotted white boxes) STM images for the (b) as-prepared and (c) base-treated samples. The corresponding slab structures are shown on the left; Bi atoms are purple, V atoms are blue, oxygen atoms are red. Further details are in ref (4).

With an understanding of bulk and surface properties, we turned to studying the impact of interfacial energetics on photoelectrochemical properties.^4^ Epitaxial BiVO_4_ photoelectrodes^44^ were used as a bridge between single-crystalline BiVO_4_ that possesses an ideal surface^43^ but cannot serve as a practical photoelectrode, and polycrystalline nanostructured BiVO_4_ that has an ill-defined surface but can serve as a high-performance photoelectrode.^45^ The epitaxial electrodes can offer well-defined surfaces and can also produce sizable photocurrents, making it possible to directly relate the variation in surface structure to the change in photoelectrochemical properties. The as-prepared epitaxial BiVO_4_ (010) electrode was slightly V-rich; a Bi-rich BiVO_4_ (010) electrode was also prepared by removing the surface V using an optimized base-etching process. The Bi-rich sample showed a considerably enhanced photocurrent, with its photocurrent onset shifted to a more negative potential (Figure 7a).

Computations were then performed to obtain an atomic-level understanding of how the observed photoelectrochemical behavior depends on the surface composition. Representative structural models for the two experimentally prepared surfaces were identified in a feedback loop by comparing simulated and measured scanning tunneling microscopy (STM) images of the two samples (Figure 7b,c). Establishing a close correspondence between the structural models in calculations with that of the epitaxial samples for the two surfaces was critical to unambiguously pinpointing how the surface composition impacts the interfacial energetics. Using the resulting structural models, our calculations revealed that an increase in the surface Bi:V ratio leads to a monotonic upward shift of the band edges. As a result, the flatband potential shifts to the negative direction and the electron–hole separation can be enhanced. These results directly connect the changes in surface composition to the experimentally observed differences in photoelectrochemical properties. Additional experimental results demonstrated that the Bi:V surface ratio of the V-rich sample increased during the photocurrent measurement at pH 9 until a stable Bi-rich surface was formed, and the photocurrent and photocurrent onset potential changed according to the Bi:V ratio during this time.

The atomic-level understanding of the surface in relation to photoelectrochemical performance gained in this study provided an invaluable basis for future studies for both computation and experiment. For BiVO_4_, many computational studies focus on the stoichiometric (010) surface (in which the Bi:V ratio is 1:1), particularly when interfaced with water.^46−49^ However, our work has identified that the relevant surface termination present in experimental conditions is actually Bi-rich, which could significantly impact the interfacial energetics upon contact with water and any secondary layers, such as protective layers or catalysts, and ultimately the reaction mechanisms for oxygen evolution.

The next important step in the investigation of interfacial properties includes the study of photoelectrode/catalyst interfaces. As a last example, we briefly describe a theoretical study that highlights the importance of investigating wet interfaces to understand the performance of catalysts and the behavior of charges at interfaces. The surface of WO_3_ is poorly catalytic for photooxidation of water, and it was experimentally demonstrated that adding IrO_2_ on WO_3_ as an oxygen evolution catalyst can considerably increase the faradaic efficiency for water oxidation.^50^ DFT calculations were used to investigate the interfacial energetics at the WO_3_/IrO_2_ junction.^51^ Since the detailed WO_3_/IrO_2_ interfacial structure was not experimentally known, a plausible coherent interfacial structure was determined with a lower formation energy than the surface energies of the most stable WO_3_ and IrO_2_ surfaces. Finding a plausible geometry involved laterally stretching the IrO_2_ slab and compressing the WO_3_ slab to overcome the 12% lattice mismatch while maintaining the same pressure on both sides of the interface.

**Figure 8:**
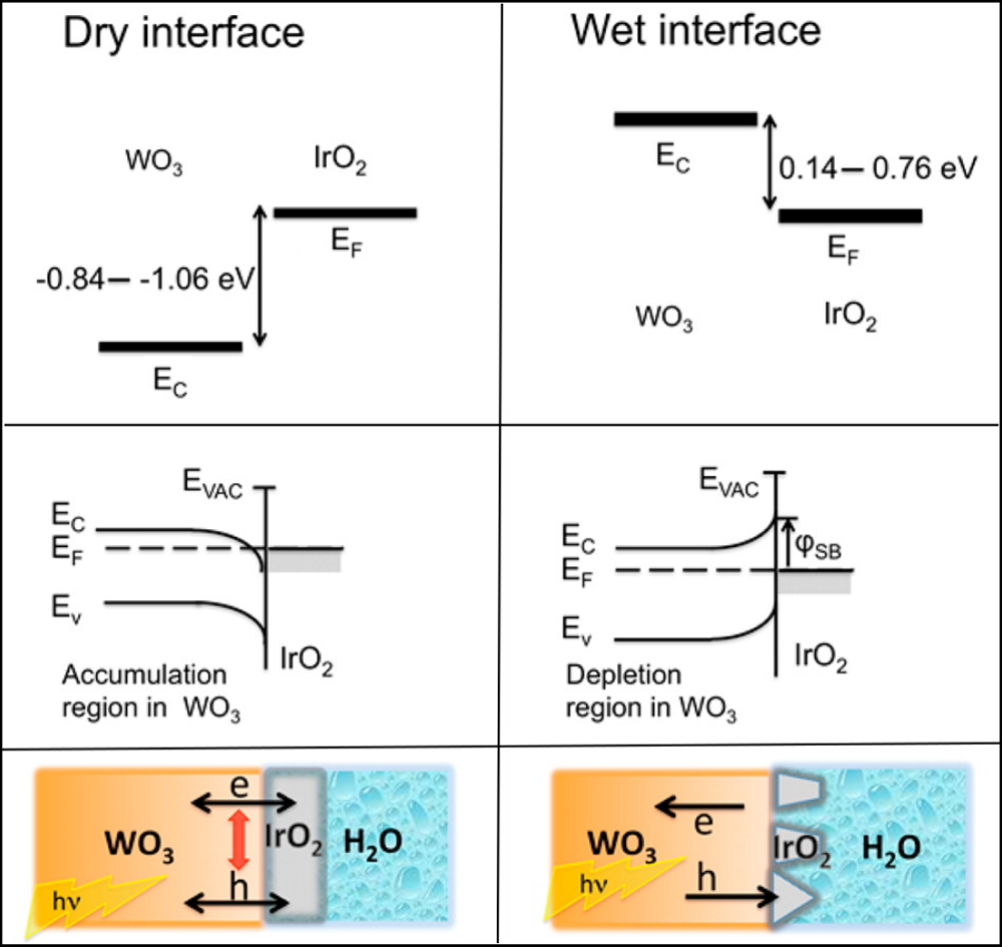
(Top) Relative alignment between the conduction band minimum of WO_3_ (*E*_C_) and the Fermi level (*E*_F_) of IrO_2_. For the dry interface (left panels), the thick lines indicate the expected numerical variations based on expected inaccuracies in the results obtained from many-body perturbation theory. For the wet interface (right panels), the thick lines represent the variation due to different water configurations at the surface. The effect of the solvent leads to a large shift of the *E*_C_ in WO_3_ relative to the Fermi level of IrO_2_. (Middle) A cartoon representation of the band-bending in the semiconductor. The dry interface leads to an ohmic contact (left panel), whereas solvation of both WO_3_ and IrO_2_ results in a Schottky barrier (φ_SB_, right panel). (Bottom) Schematic illustration of the interface. Reproduced from ref (51). Copyright 2015 American Chemical Society.

With a structural model in hand, two cases were considered. In the first case, IrO_2_ was uniformly coated over WO_3_; this led to a dry interface between WO_3_ and IrO_2_, which was determined to have an ohmic contact that leads to undesired charge accumulation at the interface (Figure 8). In the second case, IrO_2_ did not completely cover WO_3_, so water (simulated with solvation models) could be in direct contact with both WO_3_ and IrO_2_. In this case, a Schottky barrier formed, resulting in enhanced charge separation at the interface (Figure 8). This example shows how the presence of water can drastically alter the nature of photoelectrode–catalyst interactions, and it highlights an important direction of investigation for future combined theoretical and experimental investigations.

## Conclusions

We presented several studies illustrating the essential role of integrating experiment and theory to tackle complex problems such as the influence of the structure and composition of oxide materials on their photoelectrochemical properties. These studies included investigating the effects of the bulk composition, defects and dopants, and the surface and interface of photoelectrodes on photon absorption, electron–hole separation, and carrier transport, all of which affect photocurrent generation. The benefits of combining experiment and first-principles electronic structure theory in an integrated feedback loop are manifold. The first benefit is an increased knowledge of the materials physics at the microscopic scale, which leads to the ability to interpret experiments and to predict optimal materials systems, as illustrated by studies on the intercalation of N_2_ in WO_3_ that led to the identification of CO as an alternate intercalation species. Second, combining experiment and theory may uncover the interplay between different factors in our understanding of defective systems. We described how interactions between dopants and oxygen vacancies play a critical role in understanding transport properties and photocurrent generation and how introducing a defect may alter the photoelectrochemical properties in multiple interdependent ways. Third, the new microscopic insights gained from a combined study can be used to design more effective research approaches. For instance, our study on the surface composition of BiVO_4_ showed that the relevant surface termination in the experimental conditions is actually Bi-rich and not an equal ratio of Bi:V, and this will serve as a valuable guide for future studies.

The next era of research on photoelectrodes will involve studying massively complex systems, including interfaces with catalysts, spanning time and length scales that are challenging or inaccessible to experiment or computation alone. An essential component will be ensuring close correspondence between theory and experiment, particularly in the structural models and in the measured and simulated conditions. Several outstanding experimental and theoretical challenges remain in the study of photoelectrodes and water-splitting applications. Experimentally, this includes establishing well-defined and well-characterized samples, controlling only the feature of interest without unintentionally changing other features (e.g., introducing a dopant without altering morphology or surface area), disentangling surface versus bulk contributions, and probing at shorter length and time scales. Theoretically, this includes advancements in describing many-body effects and strong correlations as well as incorporating increasingly realistic and complex conditions such as temperature effects and the influence of the electrolyte and external fields. There is still much to understand about materials for photoelectrodes. For instance, charge transport and mobility are generally challenging to measure and compute. In order to achieve a quantitative and fully microscopic understanding, particularly as a function of defect concentration, further efforts are necessary for both experiment and theory. The same applies to achieving a quantitative model of the atomic and electronic structure at surfaces and interfaces. Thus, we emphasize and anticipate a renaissance in approaches to tackling the greater questions of structure–composition relationships in the bulk, at the surface, and at the interface of photoelectrode and catalyst materials through the continued integration of experiment and theory.
